# Differential Expression of Phospholipase C Epsilon 1 Is Associated with Chronic Atrophic Gastritis and Gastric Cancer

**DOI:** 10.1371/journal.pone.0047563

**Published:** 2012-10-15

**Authors:** Jun Chen, Wei Wang, Tao Zhang, Jiajia Ji, Qirong Qian, Lungeng Lu, Hualin Fu, Weilin Jin, Daxiang Cui

**Affiliations:** 1 Department of Bio-Nano Science and Engineering, Key Lab of Thin Film and Microfabrication Technology of Department of Education, Institute of Micro/Nano Science and Technology, Shanghai Jiao Tong University, Shanghai, People’s Republic of China; 2 State Key Laboratory of Oncology in South China, Sun Yat-sen University Cancer Center, Guangzhou, Guangdong, People’s Republic of China; 3 Department of Gastric & Pancreatic Surgery, Sun Yat-sen University Cancer Center, Guangzhou, Guangdong, People’s Republic of China; 4 Department of Immunology, Fujian Academy of Medical Science, Fuzhou, People’s Republic of China; 5 Department of Surgery, Changzheng Hospital affiliated to Second Military Medical University, Shanghai, People’s Republic of China; 6 Institute of Digestive Disease, The First Affiliated Hospital of Shanghai Jiao Tong University, Shanghai, People’s Republic of China; 7 School of Life Sciences and Biotechnology, Shanghai Jiao Tong University, Shanghai, People’s Republic of China; University of Navarra, Spain

## Abstract

**Background:**

Chronic inflammation plays a causal role in gastric tumor initiation. The identification of predictive biomarkers from gastric inflammation to tumorigenesis will help us to distinguish gastric cancer from atrophic gastritis and establish the diagnosis of early-stage gastric cancer. Phospholipase C epsilon 1 (PLCε1) is reported to play a vital role in inflammation and tumorigenesis. This study was aimed to investigate the clinical significance of PLCε1 in the initiation and progression of gastric cancer.

**Methodology/Principal Findings:**

Firstly, the mRNA and protein expression of PLCε1 were analyzed by reverse transcription-PCR and Western blotting in normal gastric mucous epithelial cell line GES-1 and gastric cancer cell lines AGS, SGC7901, and MGC803. The results showed both mRNA and protein levels of PLCε1 were up-regulated in gastric cancer cells compared with normal gastric mucous epithelial cells. Secondly, this result was confirmed by immunohistochemical detection in a tissue microarray including 74 paired gastric cancer and adjacent normal tissues. Thirdly, an independence immunohistochemical analysis of 799 chronic atrophic gastritis tissue specimens demonstrated that PLCε1 expression in atrophic gastritis tissues were down-regulated since PLCε1 expression was negative in 524 (65.6%) atrophic gastritis. In addition, matched clinical tissues from atrophic severe gastritis and gastric cancer patients were used to further confirm the previous results by analyzing mRNA and protein levels expression of PLCε1 in clinical samples.

**Conclusions/Significances:**

Our results suggested that PLCε1 protein may be a potential biomarker to distinguish gastric cancer from inflammation lesion, and could have great potential in applications such as diagnosis and pre-warning of early-stage gastric cancer.

## Introduction

Gastric cancer is the fourth common tumors in world. The incidence of gastric cancer is steadily increasing because of recent changes of lifestyle and diets habits in China [1]. As for gastric cancer, survival rates depend on the early diagnosis of the disease. Typically, the earlier a cancer is detected and diagnosed, the more successful the treatment will be. Therefore, it is very important to improve the rates of early diagnosis and early treatment for gastric cancer. We have tried to establish an early gastric cancer pre-warning system based on gene expression microarray analysis since 2005 [2]. Meanwhile, we also hoped to find the early gastric cancer cells *in vivo* by multi-mode targeted imaging techniques [3]–[7]. However, lacking of biomarker for gastric cancer is still the major obstacle for early diagnosis.

Nowadays, it is well accepted that *Helicobacter pylori* (*H. pylori*) plays a causal role in triggering the chronic inflammation (gastritis) leading to malignancy, and has been classified as a definite human carcinogen by International Agency of Research on Cancer (IARC) [8]–[10]. Moreover, based on the histological differences between chronic gastritis and gastric cancer, early-stage gastric cancer is often detected by endoscopy. Thus, investigation of the molecular and biological changes, including gene amplification and activations, which occur from chronic gastritis to gastric cancer, can provide some new insights into the pathology of this disease and new prognostic markers. However, up to date, there are few reports on addressing special biomarkers that can distinguish gastric inflammation from gastric tumorigenesis.

The Phosphoinositide-specific phospholipase C (PLC) represents a large family which catalyzes the hydrolysis of phosphatidylinositol 4,5-bisphosphate into two vital second messengers, diacylglycerol and inositol 1,4,5-trisphosphate, which include six families of mammalian PLC isoforms (β, γ, δ, ε, ζ and η) [11]–[13]. Among them, PLCε is a newly identified member of PLC family [12]. It is a key downstream effecter of Ras family small GTPases: Ras, Rap1, and Rap2 [12], [14], [15].

Recently, much works reported that PLCε1 played a crucial role in tumorigenesis and inflammation. PLCε1 expression has been found to correlate with human bladder cancer, and the knockdown of PLCε1 *in vitro* and *in vivo* inhibited bladder tumor growth [16], [17]. Furthermore, It has been reported that PLCε1 is over-expressed in skin cancer [18]. In PLCε^−/−^ mice, it exhibited marked resistance to tumor formation and to the 12-O-tetradecanoylphorbol-13-acetate (TPA)-induce skin inflammation [18], [19]. Subsequent studies showed that PLCε played crucial roles in spontaneous intestinal tumorigenesis with augmentation of angiogenesis and inflammation by using the adenomatous polyposis coli (Apc) ^Min/+^ mouse model [20].

Moreover, PLCε was required in activation of cytokine production in non-immune skin cells in a variety of inflammatory reactions, and this important function of PLCε was further confirmed in transgenic mice over-expressing PLCε [21], [22]. On the other hand, PLCε was required for tumor necrosis factor (TNFα)-induced chemokine (C–C motif) ligand 2 (CCL2) expression in human keratinocytes and cooperates with nuclear factor kappa B (NF-κB) pathway [23]. Such a function of PLCε in inflammation is unique among the PLC isozymes [13].

Recently, two single nucleotide polymorphism (SNP) rs2274223 and rs11187870 in PLCε1 loci have been identified as novel susceptibility locus for gastric cancer in Chinese population by genome wide gene-association analysis (GWAS) [24]–[26]. These results confirmed the association between PLCε1 and risk of gastric cancers.

In this study, we examined the expression pattern of PLCε1 in human gastric tissues including normal, atrophic gastritis, and tumor tissues, and made a hypothesis on the role of PLCε1 in chronic inflammation and tumorigenesis of gastric cancer. Compared with normal tissues, we found that PLCε1 protein was highly expressed in gastric cancer tissues, whereas it was down-regulated in atrophic gastric tissues. Our results strongly suggest that PLCε1 can be a potentially promising biomarker for distinguishing gastric cancer from atrophic gastritis and might be a potential biomarker for diagnosing early-stage gastric cancer.

## Materials and Methods

### Ethic Statements

The study was approved by the Ethics Committee of Sun Yat-sen University Cancer Center and the Ethics Committee of the First Affiliated Hospital of Shanghai Jiao Tong University, and written informed consent was obtained from each subject.

### Cell lines

The SV40-transformed immortal gastric epithelial cell GES-1 were preserved in our institute and maintained as recommended [27]. Three gastric cancer cell lines AGS, SGC7901 and MGC803 were obtained from Chinese Academy of Sciences Cell Bank of Type Culture Collection and kept in our laboratory. All cell lines were cultured in Dulbecco’s Modified Eagle Medium (DMEM) supplemented with 10% fetal calf serum (FCS) (Sigma Chemical, St. Louis, MO), 100 units/ml penicillin and 0.1 mg/ml streptomycin. The cells were maintained at 37°C in a humidified chamber with 5% CO_2_.

### RNA Extraction and Reverse Transcription-PCR

Total RNA from cells and tissues samples was extracted using the Trizol reagent (Invitrogen) according to the manufacturer’s instruction. The extracted RNA was pretreated with RNase-free DNase, and 2 µg RNA from each sample was used for cDNA synthesis primed with random hexamers. For PCR-mediated amplification of PLCε1 cDNA, an initial amplification using PLCε1-specific primers was done with a denaturation step at 95°C for 10 min followed by 30 denaturation cycles at 95°C for 60 s, primer annealing at 55°C for 30 s, and primer extension at 72°C for 30 s. On completion of the cycling steps, a final extension at 72°C for 5 min was carried out before the reaction was stopped and stored at 4°C. Expression data were normalized to the geometric mean of the housekeeping glyceraldehyde-3-phosphate dehydrogenase (GAPDH) gene to control the variability in expression levels. Reverse transcription-PCR was designed using the Primer Express Software version 3.0 (Applied Biosystems). The sequences of the sense and antisense primers were as follows: 5′-GCAAGAAGTGGCCTTCTCAG-3′ (F) and 5′-GAACTTGAAGGGAGGGCATT -3′ (R) for PLCε1, 5′-GCTCCTCCTGAGCGCAAG-3′ (F) and 5′-CATCTGCTGGAAGGTGGACA - 3′(R) for GAPDH.

### Western Blotting

Cells were harvested in sampling buffer [62.5 mmol/L Tris-HCl (pH 6.8), 2% SDS, 10% glycerol, and 5% 2-h-mercaptoethanol]. All fresh tissues were grounded to powder in liquid nitrogen and then lysed with the sampling buffer. Protein concentration was determined by Bradford assay (Bio-Rad Laboratories). Equal amounts of proteins were applied to 9% polyacrylamide SDS gels (SDS-PAGE), separated electrophoretically, and transferred onto polyvinylidene fluoride membranes (Amersham Pharmacia Biotech). The membrane was incubated with anti- PLCε1 rabbit antibody (1∶250; Sigma). PLCε1 expression was detected with horseradish peroxidase-conjugated goat anti-rabbit IgG (1∶2,000; Amersham Pharmacia Biotech) and an enhanced chemiluminescence kit (Amersham Pharmacia Biotech) according to the manufacturer’s instructions. β-actin detected with an anti-β-actin rabbit antibody (1∶1,000 dilution; Sigma) was used as the loading control.

### Patients, Tissue Specimens and Tissue Microarray

Patients with atrophic gastritis and cancer were selected from patients who were scheduled for upper gastrointestinal endoscopy for routine screening for gastric cancer at the First Affiliated Hospital of Shanghai Jiao Tong University and Sun Yat-sen University Cancer Center from January 2003 to December 2009. We excluded patients who had received anti-ulcer agents or antibiotics for up to two months before the examination and those who had histories of gastric cancer, gastric or duodenal ulcer, or gastric surgery. Hematoxylin and eosin (H&E) staining on patient tissues was done to determine histopathologic features.

The degree of atrophy was classified into three levels of severity, according to the appearance of mucosal folds and vasculature, as follows: mild (transparent fine blood vessels and yellowish discoloration limited to the lower body, with thick mucosal folds), moderate (clearly transparent blood vessels and yellow-grayish discoloration up to the middle and upper body, with thinned and narrowed mucosal folds), and severe (clearly transparent large blood vessels and graygreenish discoloration up to the upper body, with disappearance of mucosal folds on air insufflation). The use of these criteria is justified by the previous reports on the consistency between endoscopy and histological findings for atrophic gastritis [28], [29]. When both endoscopy and histological findings were confirmed, 799 atrophic gastritis patients including 281 mild, 265 moderate and 253 severe, respectively, were enrolled into our study. The 799 patients were included 439 male and 320 female, with a media age of 35.6 years (range, 18–55 years).

Each tumor sample was assigned a histological grade based on the World Health Organization (WHO) classification criteria. All gastric cancer patients were staged using the 7th edition of the International Union Against Cancer (UICC) Tumor-Node-Metastasis (TNM) staging system. Patients with cancer were enrolled into our study when their diagnosis was histologically confirmed. The normal tissues were eligible if their endoscopic diagnosis was normal. After screening H&E-stained slides for optimal tumor tissue and normal tissue adjacent to tumor with a distance of 10 cm from the tumor, we constructed TMA slides with 74 matched pairs of tumor tissues and adjacent to the tumor tissues (collaborating with Shanghai Biochip, Shanghai, China).The 74 gastric cancer patients (age range = 40–84 years; average age = 63.77 years; 23 female and 35 male) included 36 early cases (pTNM stage I = 11, pTNM stage II = 25) and 36 advanced cases (pTNM stage III = 32, pTNM stage IV = 6). Among these, 6 (0.81%) were classified as well differentiated adenocarcinoma, 40 (54.1%) as moderately differentiated, and 25 (33.8%) as poorly differentiated adenocarcinoma.

### Immunohistochemistry

Immunohistochemical analysis was used to determine the expression pattern of PLCε1 protein in 74 matched human gastric cancer tissues and 799 atrophic gastritis samples. In brief, paraffin embedded specimens were cut into 4 µm sections and baked at 60°C for 2 h followed by deparaffinization with xylene and rehydrated. The sections were submerged into EDTA antigenic retrieval buffer and microwaved for antigenic retrieval, after which they were treated with 3% hydrogen peroxide in methanol to quench endogenous peroxidase activity, followed by incubation with 1% bovine serum albumin to block nonspecific binding. Sections were incubated with rabbit anti- PLCε1 (1∶50; Sigma) overnight at 4°C. Normal goat serum was used as a negative control. After washing, tissue sections were treated with biotinylated anti-rabbit secondary antibody (Zymed) followed by further incubation with streptavidin-horseradish peroxidase complex (Zymed). Tissue sections were then immersed in 3, 3′-diaminobenzidine and counterstained with 10% Mayer’s hematoxylin, dehydrated, and mounted.

### Scoring of PLCε1 Immunohistochemistry

PLCε1 immunoreactivity was evaluated independently by 3 pathologists (W.W., Q.Q. and L.L.) who were blinded to patient outcomes. The percentage of positively stained cells and intensity of staining cells was determined by each observer, and the average of 3 scores was calculated. We randomly selected 10 high-power fields (magnification, ×400; 100 cells per high-power field) and counted 1000 cells. When the mean of percentage of PLCε1-positive cells is close to 0% or 100%, the standard deviation (SD) is close to 0; and, when the mean is approximately 50%, the SD is approximately 5%. Thus, the SD does not increase with the mean. In this study, PLCε1 expression was graded as follows: tissue with positive cytoplasmic staining in ≤25% of cells was graded negative, and tissue with positive cytoplasmic staining in >25% of cells was graded positive [30].

### Statistical Analysis

All statistical analyses were carried out using the SPSS 17.0 statistical software package. The wilcoxon test method was used to analyze those matched gastric tumors tissues in the tissue microarray. The 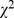 test and Fisher’s exact tests used to analyze those gastric cancer and severe atrophic gastritis, and also were performed to determine the significance of the relationship between PLCε1 expression and clinicopathologic characteristics. Each experiment was performed independently at least twice with similar results. *p*<0.05 in all cases was considered statistically significant.

## Results

### Up-regulated Expression of PLCε1 in Gastric Cancer Cell Lines

**Figure 1:**
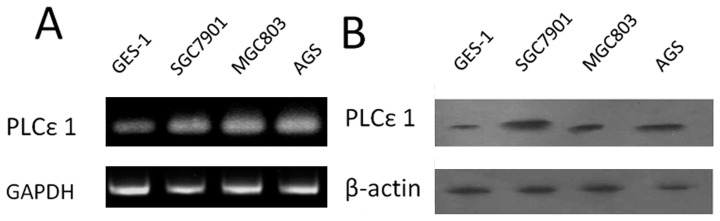
Expression analysis of PLCε1 protein and mRNA in gastric normal and cancer cell lines. A, expression of PLCε1 mRNA in gastric normal cell line GES-1and cancer cell lines SGC7901, MGC803 and AGS by reverse transcription-PCR. B, expression of PLCε1 protein gastric normal cell lines GES-1 and cancer cell lines SGC7901, MGC803 and AGS by Western blotting.

Firstly, we detected the mRNA and protein expression of PLCε1 in different gastric cell lines (Figure 1). Reverse transcription-PCR analysis and Western blotting analysis were conducted on these samples derived from normal gastric mucous cells GES-1 and three gastric cancer cell lines AGS, SGC7901 and MGC803. We found that all gastric cancer cell lines revealed higher PLCε1 expression than that in normal gastric cell controls at both mRNA and protein levels.

### PLCε1 is up-regulated in Gastric Cancer Lesions but down-regulated in Atrophic Gastritis Lesions

**Figure 2:**
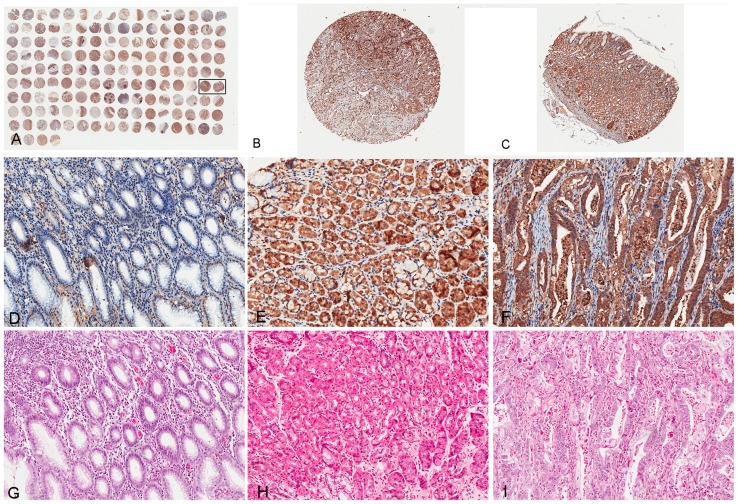
Immunohistochemical detection of PLCε1 protein in gastric cancer and atrophic gastritis patients. Representative images from Immunohistochemical analysis of 74 archived gastric cancer and 799 atrophic gastritis cases. A, overview of the tissue microarray by immunostaining of antibody PLCε1. Boxed biopsies are shown in detail in (B and C). B and C are matched tumor and adjacent normal tissues from same patient. The patient is pTNM stage III with poorly differentiated adenocarcinoma. Both tumor (B) and adjacent normal (C) cells are positive for PLCε1. Representative immunohistochemical (D)/histological (G) observations in a atrophic gastritis tissue, Representative immunohistochemical (E)/histological (H)observations in a gastric normal tissue, Representative immunohistochemical (F)/histological (I) observations in a gastric cancer tissue. (Original magnification, ×2 for B and C, ×200 for D, E, F, G, H and I).

Then, we investigated the role of PLCε1 in both tumor and normal tissues in a gastric cancer tissue microarray. As shown in Figure 2, we observed that both tumor and normal tissues demonstrated the positive expression of PLCε1. However, as shown in Table 1, tissue microarray data analysis had shown that the percentage of PLCε1 positive expression in tumor tissues is significantly higher than that in adjacent normal tissues by wilcoxon test analysis (73.0% versus 20.3%, *p*<0.01).

**Table 1:** The expression of PLCε1 protein in gastric cancer tissue and adjacent normal tissue.

PLCε1 protein staining (N = 74)	Negative	Positive	High	Low		Equality	*p* value (wilcoxon test)
Gastric cancer tissue	0 (0%)	74 (100%)					
Adjacent normal tissue	0 (0%)	74 (100%)					
Tumor tissue versus adjacent normal tissue			54 (73.0%)		15 (20.3%)	5 (6.70%)	*p*<0.001

To determine PLCε1 expression in inflammation environment, we extended PLCε1 immunohistochemical analysis using another independent set of paraffin-embedded tissue sections of 799 atrophic gastritis tissue specimens. The immunohistochemical results showed that PLCε1 expression was positive in 275 (37.0%) atrophic gastritis including 106 (37.7%) mild atrophic gastritis, 98 (26.0%) moderate atrophic gastritis and 71 (28.1%) in severe atrophic, respectively. Moreover, the PLCε1 expression was correlated with the degree of atrophy (*p* = 0.036) (see Table 2). Therefore, compared with tumor and normal tissues, atrophic gastritis samples had decreased PLCε1 expression (*p*<0.001) (see Table 2).

**Table 2:** The expression of PLCε1 protein in gastric cancer, normal and atrophic gastritis patients.

PLCε1 protein staining (N = 947)	Negative	Positive	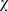 ^2^ test	Fisher’s exact test
Gastric cancer	0(0%)	74 (100%)		
Normal	0(0%)	74 (100%)		
Atrophic gastritis	524(63.0%)	275(37.0%)	*p*<0.001	*p*<0.001
Mild atrophic	175(62.3%)	106(37.3%)		
Moderate atrophic	167(74.0%)	98(26.0%)		
Severe atrophic	182(71.9%)	71(28.1%)	*p* = 0.036	*p* = 0.034

**Figure 3:**
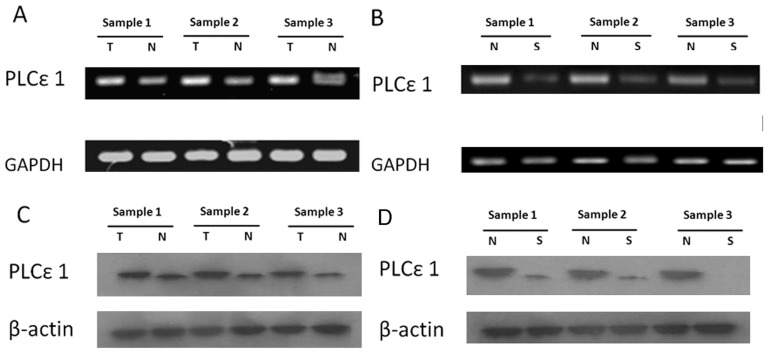
Expression analysis of PLCε1 mRNA and protein in gastric cancer and atrophic gastritis patients. A and C, expression of PLCε1 mRNA and protein in each of the gastric tumors (T) and gastric adjacent normal tissues (N) paired from the same patient by reverse transcription-PCR and western blot. B and D, expression of PLCε1mRNA and protein in each of the severe atrophic gastritis (S) and gastric adjacent normal tissues (N) paired from the same patient by reverse transcription-PCR and Western blotting.

We then confirmed these immunohistochemical tissue microarray results by reverse-transcription-PCR and Western blotting analysis of three paired of gastric cancer and adjacent normal tissues and three paired of severe atrophic gastritis and adjacent normal tissues, which were taken from the same patient. Compared with adjacent normal tissues, three examined gastric tumors displayed up-regulation of PLCε1 expression at both mRNA and protein levels, but all of severe atrophic gastritis tissues had lower expression of PLCε1(see Figure.3). In agreement with the results of immunohistochemical tissue microarray analysis, the paired analysis also showed PLCε1 was over-expressed in tumor, but down-regulated in severe atrophic gastritis in comparison with the matched adjacent normal tissues.

These observations suggested that PLCε1 expression status was very different in the context of inflammation versus tumorigenesis. Our results highly suggest that our results highly suggest that the PLCε1 protein could be a potential biomarker of gastric cancer.

### Correlations between PLCε1 and Clinicopathologic Features

There is no statistically significant difference of PLCε1 expression in clinicopathologic data, such as age, sex, tumor size, classification, clinical stage, between patients at different stages of gastric cancer in the tissue microarray (shown in Table S1).

Particularly, as shown in Figure 2, PLCε1 protein expression in cytoplasm was frequently detected in all tumor tissues, normal tissues but less frequently in atrophic gastritis tissues.

**Figure 4:**
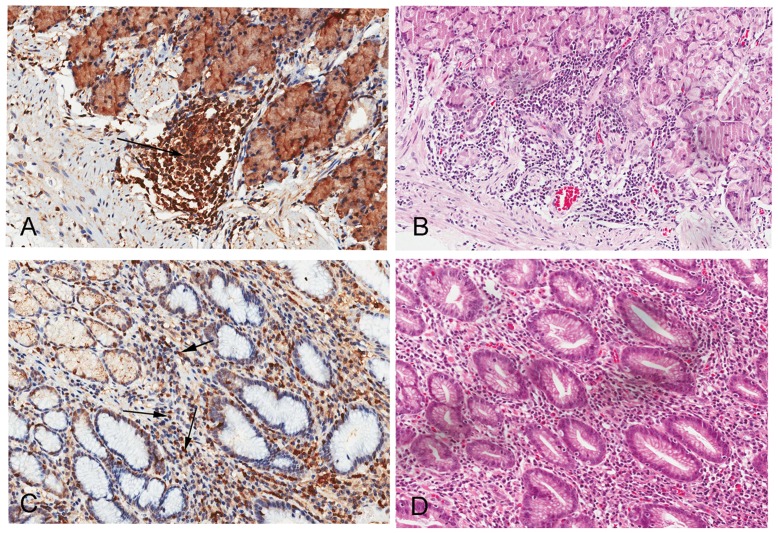
PLCε1 is positively expressed in lymphocytes in normal and atrophic gastritis tissues, respectively. Representative immunohistochemical (A)/histological (B) observations in a gastric normal tissue, Representative immunohistochemical (C)/histological (D) observations in a atrophic gastritis tissue. Arrows indicate positively stained immune cells (Original magnification, ×200).

However, as shown in Figure 4, in the tissue microarray, positive PLCε1 expression were observed in inflammatory cells and lymphocytes in 13 (48.1%) adjacent normal tissue samples from lymphnode metastases (N0) gastric cancer patients (shown in Figure 4A). Moreover, in severe atrophic gastritis tissues, 6 (2.37%) samples showed positive PLCε1 expression in inflammatory cells or lymphatic tissues (Figure 4B). Thus, these results imply that the expression of PLCε1 may be associated with inflammatory cells invasion during tumorigenesis and tumor metastases.

## Discussion

Here, our study firstly demonstrated that PLCε1 was up-regulated in human gastric tumor tissues, but was down-regulated in inflammation environment, compared with normal tissues. The differential expression of PLCε1 in different tissues highly suggested that the expression of PLCε1 could play a key role in inflammation and tumorigenesis.

Inflammation is recognized as a vital constituent of the local environment sustaining tumor development, including initiation, promotion, malignant conversion, invasion, and metastasis [31]. A causal relationship between gastric tumor promotion and inflammation has been supported from the observations that the inflammation caused by *Helicobacter pylori* infection can increase the risk of tumor promotion [8]–[10]. Recently, numerous reports have shown that PLCε1 expression levels are highly associated with inflammation and tumorigenesis [19]–[21], [23]. However, the molecular mechanism underlying the biological significance of PLCε1 in gastric cancer development and progression remains obscure. Our study has provided evidence that PLCε1 up-regulation might be important in the progression of gastric cancer. Particularly, its down-regulation in inflammation environment, highly suggested that PLCε1 may be an important biomarker, and may be used to distinguish gastric cancer status from atrophic lesion stage.

Moreover, in our work, PLCε1 protein can be detected in inflammatory cells and lymphocyte tissues in some adjacent normal tissues at N0 degree and some severe atrophic gastritis tissues. In mammals, PLCε1 is reported to be expressed in non-immune cells such as epidermal keratinocytes, dermal fibroblasts, and epithelial cells, but not in immune cells such as lymphocytes, granulocytes, macrophages, and dendritic cells [21], [32], [33]. Our results suggest that the function of PLCε1 protein in gastric cancer may be linked to its expression in the immune system.

According to these available results, we proposed a possible mechanism of PLCε1 protein functioning in tumorigenesis of gastric cancer: PLCε1 protein as an effecter of Ras and Rap small GTPases [12], [14], [15] is at first expressed in normal gastric mucous tissues. In the course of progression of atrophic gastric lesion, PLCε1 protein expression is decreased to prevent early inflammation infiltration, according to PLCε^−/−^ mice exhibits marked resistance to the TPA-induce skin inflammation [18], [19]. With increasing time and degree of inflammation of atrophic gastric lesion, PLCε1 protein is gradually expressed in immune cells because PLCε was required in a variety of inflammatory reactions [21], [22], which may finally induce the formation of gastric cancer. Simultaneously, PLCε1 protein gets higher expression in gastric cancer cells because PLCε over-expression can promote intestinal tumorigenesis in Apc^Min/+^ mice [20].

In summary, up-regulation of PLCε1 expression in gastric cancer but down-regulation in atrophic gastritis was observed by our study. The importance role of PLCε1 in gastric cancer is further highlighted by our finding of its inverse correlation with chronic atrophic gastritis. These results not only suggest that PLCε1 may be used as a prognostic indicator to distinguish gastric cancer from atrophic gastritis, but also warrant further studies to establish a possible link between the biological function of PLCε1 and the pathogenesis of gastric cancer. Our studies also show that PLCε1 may have an important role in tumorigenesis of gastric cancer, especially at early stage, and might represent a novel marker for diagnosing early-stage gastric cancer.

## Supporting Information

Table S1 The relationship between PLCε1 expression and clinicopathologic characteristics.
(DOC)

## References

[1] Parkin DM, Bray F, Ferlay J, Pisani P (2005) Global Cancer Statistics, 2002. CA: A Cancer Journal for Clinicians 55: 74–108. 15761078 10.3322/canjclin.55.2.74

[2] Cui DX, Zhang L, Yan XJ, Zhang LX, Xu JR, et al (2005) A microarray-based gastric carcinoma prewarning system. World Journal of Gastroenterology 11: 1273–1282. 15761963 10.3748/wjg.v11.i9.1273 PMC4250672

[3] Li Z, Huang P, Zhang X, Lin J, Yang S, et al (2009) RGD-Conjugated Dendrimer-Modified Gold Nanorods for in Vivo Tumor Targeting and Photothermal Therapy†. Molecular Pharmaceutics 7: 94–104. 10.1021/mp9001415 19891496

[4] Kong Y, Chen J, Gao F, Li W, Xu X, et al (2010) A Multifunctional Ribonuclease-A-Conjugated CdTe Quantum Dot Cluster Nanosystem for Synchronous Cancer Imaging and Therapy. Small 6: 2367–2373. 20927799 10.1002/smll.201001050

[5] Chen L, Bao C-C, Yang H, Li D, Lei C, et al (2011) A prototype of giant magnetoimpedance-based biosensing system for targeted detection of gastric cancer cells. Biosensors and Bioelectronics 26: 3246–3253. 21239159 10.1016/j.bios.2010.12.034

[6] He M, Huang P, Zhang C, Hu H, Bao C, et al (2011) Dual Phase-Controlled Synthesis of Uniform Lanthanide-Doped NaGdF4 Upconversion Nanocrystals Via an OA/Ionic Liquid Two-Phase System for In Vivo Dual-Modality Imaging. Advanced Functional Materials 21: 4470–4477.

[7] Huang P, Li Z, Lin J, Yang D, Gao G, et al (2011) Photosensitizer-conjugated magnetic nanoparticles for in vivo simultaneous magnetofluorescent imaging and targeting therapy. Biomaterials 32: 3447–3458. 21303717 10.1016/j.biomaterials.2011.01.032

[8] Parsonnet J, Friedman GD, Vandersteen DP, Chang Y, Vogelman JH, et al (1991) Helicobacter pylori Infection and the Risk of Gastric Carcinoma. New England Journal of Medicine 325: 1127–1131. 1891020 10.1056/NEJM199110173251603

[9] Humans IWGotEoCRt (1994) Schistosomes, liver flukes and Helicobacter pylori. PMC7681621 7715068

[10] Uemura N, Okamoto S, Yamamoto S, Matsumura N, Yamaguchi S, et al (2001) Helicobacter pylori Infection and the Development of Gastric Cancer. New England Journal of Medicine 345: 784–789. 11556297 10.1056/NEJMoa001999

[11] Rhee SG, Bae YS (1997) Regulation of Phosphoinositide-specific Phospholipase C Isozymes. Journal of biological chemistry 272: 15045–15048. 9182519 10.1074/jbc.272.24.15045

[12] Song C, Hu C-D, Masago M, Kariya K-i, Yamawaki-Kataoka Y, et al (2001) Regulation of a Novel Human Phospholipase C, PLCepsilon, through Membrane Targeting by Ras. J Biol Chem 276: 2752–2757. 11022048 10.1074/jbc.M008324200

[13] Suh P-G, Park J-I, Manzoli L, Cocco L, Peak J, et al (2008) Multiple roles of phosphoinositide-specific phospholipase C isozymes. BMB Reports 41: 415–434. 18593525 10.5483/bmbrep.2008.41.6.415

[14] Bunney TD, Harris R, Gandarillas NL, Josephs MB, Roe SM, et al (2006) Structural and Mechanistic Insights into Ras Association Domains of Phospholipase C Epsilon. Molecular cell 21: 495–507. 16483931 10.1016/j.molcel.2006.01.008

[15] Bunney TD, Katan M (2006) Phospholipase C epsilon: linking second messengers and small GTPases. Trends in cell biology 16: 640–648. 17085049 10.1016/j.tcb.2006.10.007

[16] Cheng H, Luo C, Wu X, Zhang Y, He Y, et al (2011) shRNA Targeting PLCe1 Inhibits Bladder Cancer Cell Growth In Vitro and In Vivo. Urology 78: 474.e477–474.e411. 10.1016/j.urology.2011.03.014 21705050

[17] Ou L, Guo Y, Luo C, Wu X, Zhao Y, et al (2010) RNA interference suppressing PLCE1 gene expression decreases invasive power of human bladder cancer T24 cell line. Cancer genetics and cytogenetics 200: 110–119. 20620593 10.1016/j.cancergencyto.2010.01.021

[18] Bai Y, Edamatsu H, Maeda S, Saito H, Suzuki N, et al (2004) Crucial Role of Phospholipase Ce in Chemical Carcinogen-Induced Skin Tumor Development. Cancer research 64: 8808–8810. 15604236 10.1158/0008-5472.CAN-04-3143

[19] Ikuta S, Edamatsu H, Li M, Hu L, Kataoka T (2008) Crucial Role of Phospholipase Cε in Skin Inflammation Induced by Tumor-Promoting Phorbol Ester. Cancer research 68: 64–72. 18172297 10.1158/0008-5472.CAN-07-3245

[20] Li M, Edamatsu H, Kitazawa R, Kitazawa S, Kataoka T (2009) Phospholipase Cε promotes intestinal tumorigenesis of ApcMin/+ mice through augmentation of inflammation and angiogenesis. Carcinogenesis 30: 1424–1432. 19458037 10.1093/carcin/bgp125

[21] Hu L, Edamatsu H, Takenaka N, Ikuta S, Kataoka T (2010) Crucial Role of Phospholipase Cε in Induction of Local Skin Inflammatory Reactions in the Elicitation Stage of Allergic Contact Hypersensitivity. The journal of immunology 184: 993–1002. 20007527 10.4049/jimmunol.0901816

[22] Takenaka N, Edamatsu H, Suzuki N, Saito H, Inoue Y, et al (2011) Overexpression of phospholipase Cε in keratinocytes upregulates cytokine expression and causes dermatitis with acanthosis and T-cell infiltration. European Journal of Immunology 41: 202–213. 21182091 10.1002/eji.201040675

[23] Harada Y, Edamatsu H, Kataoka T (2011) PLCε cooperates with the NF-κB pathway to augment TNFα-stimulated CCL2/MCP1 expression in human keratinocyte. Biochemical and Biophysical Research Communications 414: 106–111. 21951843 10.1016/j.bbrc.2011.09.032

[24] Wang M, Zhang R, He J, Qiu L, Li J, et al (2012) Potentially Functional Variants of <italic>PLCE1</italic> Identified by GWASs Contribute to Gastric Adenocarcinoma Susceptibility in an Eastern Chinese Population. Plos One 7: e31932. 22412849 10.1371/journal.pone.0031932 PMC3295761

[25] Abnet CC, Freedman ND, Hu N, Wang Z, Yu K, et al (2010) A shared susceptibility locus in PLCE1 at 10q23 for gastric adenocarcinoma and esophageal squamous cell carcinoma. Nat Genet 42: 764–767. 20729852 10.1038/ng.649 PMC2947317

[26] Wang L-D, Zhou F-Y, Li X-M, Sun L-D, Song X, et al (2010) Genome-wide association study of esophageal squamous cell carcinoma in Chinese subjects identifies susceptibility loci at PLCE1 and C20orf54. Nat Genet 42: 759–763. 20729853 10.1038/ng.648

[27] Zhang Z, Li Z, Gao C, Chen P, Chen J, et al (2008) miR-21 plays a pivotal role in gastric cancer pathogenesis and progression. Lab Invest 88: 1358–1366. 18794849 10.1038/labinvest.2008.94

[28] Inoue M, Kobayashi S, Matsuura A, Hamajima N, Tajima K, et al (1998) Agreement of endoscopic findings and serum pepsinogen levels as an indicator of atrophic gastritis. Cancer epidemiology, biomarkers & prevention : a publication of the American Association for Cancer Research, cosponsored by the American Society of Preventive Oncology 7: 261–263. 9521444

[29] Inoue M, Tajima K, Matsuura A, Suzuki T, Nakamura T, et al (2000) Severity of chronic atrophic gastritis and subsequent gastric cancer occurrence: a 10-year prospective cohort study in Japan. Cancer Letters 161: 105–112. 11078919 10.1016/s0304-3835(00)00603-0

[30] Qian Y-B, Zhang J-B, Wu W-Z, Fang H-B, Jia W-D, et al (2006) P48 is a predictive marker for outcome of postoperative interferon-α treatment in patients with hepatitis B virus infection-related hepatocellular carcinoma. Cancer 107: 1562–1569. 16948122 10.1002/cncr.22206

[31] Grivennikov SI, Greten FR, Karin M (2010) Immunity, Inflammation, and Cancer. Cell 140: 883–899. 20303878 10.1016/j.cell.2010.01.025 PMC2866629

[32] Boyer O, Benoit G, Gribouval O, Nevo F, Pawtowski A, et al (2010) Mutational analysis of the PLCE1 gene in steroid resistant nephrotic syndrome. Journal of Medical Genetics 47: 445–452. 20591883 10.1136/jmg.2009.076166

[33] Hinkes B, Wiggins RC, Gbadegesin R, Vlangos CN, Seelow D, et al (2006) Positional cloning uncovers mutations in PLCE1 responsible for a nephrotic syndrome variant that may be reversible. Nat Genet 38: 1397–1405. 17086182 10.1038/ng1918

