# Supplementary material for: Differential Expression of Phospholipase C Epsilon 1 Is Associated with Chronic Atrophic Gastritis and Gastric Cancer
Source: PLoS One. 2012 Oct 15;7(10):e47563. doi: 10.1371/journal.pone.0047563 (PMC3471869; doi:10.1371/journal.pone.0047563)
Supplement: Table S1 — The relationship between PLCε1 expression and clinicopathologic characteristics. (DOC) [file pone.0047563.s001.doc]

Differential expression of Phospholipase C epsilon 1 is associated with chronic atrophic gastritis and gastric cancer

Jun Chen^1^, Wei Wang^2,3^, Tao Zhang^4^, Jiajia Ji^1^, Qirong Qian^5^, Lungeng Lu^6^, Hualin Fu^1^, Weilin Jin^1,7^, Daxiang Cui^1*^

**Supporting information**

**Table S1.** The relationship between PLCε1 expression and clinicopathologic characteristics

| Characteristics | PLCε1 | | $\chi$^2^ test | Fisher’s exact test |
| --- | --- | --- | --- | --- |
|  | High | Low |  |  |
|  | No.case (%) | No.case (%) |  |  |
| age (years) |  |  |  |  |
| ≥ 60 | 41(75.9%) | 9(60.0%) | 0.222 | 0.326 |
| ＜ 60 | 13(24.1%) | 6(40.0%) |  |  |
| sex |  |  |  |  |
| male | 35(64.8%) | 11(73.3%) | 0.536 | 0.758 |
| female | 19(35.2%) | 4(26.7%) |  |  |
| clinical stage |  |  |  |  |
| 1 | 7(13.0%) | 3(20.0%) | 0.851 | 0.865 |
| 2 | 19(35.2%) | 4(26.7%) |  |  |
| 3 | 23(42.6%) | 7(46.7%) |  |  |
| 4 | 5(9.3%) | 1(6.7%) |  |  |
| tumor size |  |  |  |  |
| ≥ 5cm | 36(66.7%) | 8(53.3%) | 0.342 | 0.375 |
| ＜ 5cm | 18(33.3%) | 7(46.7%) |  |  |
| T classification |  |  |  |  |
| T1 | 3(5.6%) | 1(6.7%) | 0.996 | 1 |
| T2 | 10(18.5%) | 3(20.0%) |  |  |
| T3 | 33(61.1%) | 9(60.0%) |  |  |
| T4 | 8(14.8%) | 2(13.3%) |  |  |
| N classification |  |  |  |  |
| N0 | 21(38.9%) | 3(20.0%) | 0.531 | 0.526 |
| N1 | 8(14.8%) | 3(20.0%) |  |  |
| N2 | 11(20.4%) | 3(20.0%) |  |  |
| N3 | 14(25.9%) | 6(40.0%) |  |  |
| metastasis |  |  |  |  |
| no | 50(92.6%) | 13(86.7%) | 0.471 | 0.604 |
| yes | 4(7.4%) | 2(13.3%) |  |  |
| pathologic differentiation |  |  |  |  |
| 1 | 5(9.3%) | 1(6.7%) | 0.916 | 1 |
| 2 | 30(55.6%) | 8(53.3%) |  |  |
| 3 | 19(35.2%) | 6(40.0%) |  |  |
| venous invasion |  |  |  |  |
| no | 53(98.1%) | 15(100.0%) | 0.595 | 1 |
| yes | 1(1.9%) | 0(0%) |  |  |
| lymphatic invasion |  |  |  |  |
| no | 21(38.9%) | 3(20.0%) | 0.174 | 0.229 |
| yes | 33(61.1%) | 12(80.0%) |  |  |
